# Risk and prognosis of second cutaneous melanoma after radiotherapy for breast cancer: A population-based analysis

**DOI:** 10.17305/bb.2023.10029

**Published:** 2024-08-01

**Authors:** Tianxin Luo, Yani Zhang, Tianliang Chen, Yanxia Cai, Zheng Yang

**Affiliations:** 1 School of Public Health, Guangdong Medical University, Dongguan, China; 2 Department of Dermatology, Guangdong Medical Affiliated Hospital, Zhanjiang, China

**Keywords:** Breast cancer (BC), second primary malignancies, cutaneous melanoma (CM), radiation therapy (RT), survival outcomes, the Surveillance, Epidemiology, and End Results (SEER)

## Abstract

Radiation therapy (RT), a primary treatment for breast cancer (BC), may be associated with increased non-BC tumor risk. We aimed to examine second cutaneous melanoma (SCM) risk in BC patients who underwent RT and to assess their survival outcomes. Data from 520,977 BC patients diagnosed between 1973–2018 were collected from the Surveillance, Epidemiology, and End Results (SEER) database. Cumulative SCM incidence was estimated using the Fine-Gray competing risk model. Poisson regression analysis was conducted to calculate the standardized incidence ratio (SIR) and estimate the SCM relative risk (RR) in patients who underwent RT compared to those who did not. Overall survival (OS) and cancer-specific survival (CSS) were assessed using the Kaplan–Meier method. Among the 520,977 BC patients, 243,676 (46.8%) underwent surgery and RT, while 277,301 (53.2%) only underwent surgery. Our results suggest that BC patients receiving RT had a higher SCM risk than those who did not (hazard ratio [HR] 1.40; 95% confidence interval [CI] 1.30–1.51; *P* < 0.001). SCM incidence was also higher in BC patients treated with RT than in the general US population (SIR 1.12; 95% CI 1.05–1.19; *P* < 0.05). However, SCM patients who received RT had a significantly higher 10-year survival rate than those who did not receive RT (14.90% vs 5.94%; *P* < 0.001). No significant difference was found in 10-year OS or 5-year CSS between SCM following RT and only primary cutaneous melanoma (OPCM), but SCM patients who did not receive RT had a significantly lower 10-year OS, with no significant difference in CSS. This study suggests an increased SCM likelihood in BC patients due to RT, although the overall risk is minimal.

## Introduction

Breast cancer (BC) has the highest incidence rate among women in most countries [1]. However, advancements in modern diagnostic and therapeutic techniques have significantly improved the survival rate of BC patients. The 5- and 10-year survival rates are nearly 90% and 60%, respectively [2–4]. As BC survival rates continue to rise, concerns about patients’ quality of life have grown more pressing. One possible contributing factor to the reduced quality of life may be the diverse treatment methods used. Among BC patients, radiation therapy (RT) is a popular choice because it can effectively eradicate tumor cells and prevent them from metastasizing. However, ionizing radiation can harm vital molecules, such as deoxyribonucleic acid (DNA) and proteins, leading to significant harm to the patient. There is substantial evidence linking radiation exposure to cancer development [5, 6]. It has also been suggested that radiation exposure to peripheral tissues during the treatment of BC may lead to the development of a second cancer through a similar mechanism [7].

Previous epidemiological studies have provided evidence of the association between BC and cutaneous melanoma (CM). According to Goggins et al. [8], female patients with BC treated with RT had a 42% higher risk of developing melanoma. Another study by Jeyakumar et al. [9] concluded that the standardized incidence ratio (SIR) for BC patients with second CM (SCM) ranged from 1.16–5.13. However, the issue of increased risk of developing SCM after RT remains inadequately explored. Previous population-based retrospective studies have provided limited and conflicting findings on this topic. Most of the studies have relied on the SIR to determine the risk of developing SCM in BC survivors, but the implications of SCM regarding prognosis and care after RT have not been extensively discussed. Therefore, this study was conducted to investigate the risk of developing SCM from RT and evaluate the survival outcomes of patients.

## Materials and methods

### Data source and study population

Access to nine registries from the Surveillance, Epidemiology, and End Results (SEER) database was granted to collect data on patients diagnosed with first primary BC between January 1975 and December 2018. Patients with a confirmed pathological diagnosis of BC (C50.0–C50.9) were initially included, adhering to the coding guidelines of the International Classification of Diseases in Oncology, Third Edition (ICD-O-3) as the standard. The selection of tumor stage was restricted to local and regional cases. Exclusion criteria comprised patients without surgical intervention and in whom BC was not their first primary cancer, age under 20 years, distant metastases, survival less than a year after treatment initiation, and incomplete or missing information regarding RT, surgery, age, tumor stage, race, and survival status or follow-up information. CM patient data were sourced from the same database.

### Interventions and outcomes

Patients with primary BC were categorized into two groups based on whether they underwent RT. The RT group included patients who underwent surgery and (adjuvant) RT, while the non-RT (NRT) group comprised those only receiving surgery. The SEER database collects information on initial treatment but lacks radiation dose data.

The primary outcome of interest in this study was the occurrence of SCM, defined as tumor developing in BC patients at least one-year post-initial treatment. Endpoint events were the occurrence of a positive event, death from any cause, or termination of follow-up, whichever occurred first. Secondary outcomes were overall survival (OS) and cancer-specific survival (CSS) in SCM. The time of diagnosis of CM served as the initial event, while any cases resulting in death from any cause during the follow-up period were considered the endpoint. Data without exact survival times, including information on patients who survived until the end of follow-up, were censored. Unlike OS, which does not provide specific information on the cause of death, CSS defines the cause of death according to CM etiology. Non-SCM deaths were considered competing risk events for death, while data on living patients were censored.

### Ethical statement

This study, based on the publicly available SEER database, did not require the Institutional Review Board’s approval.

### Statistical analysis

Baseline patient information was collected and analyzed using R software version 4.2.3. Categorical variables were presented by frequency or percentage. The differences between groups were compared using the chi-square test, with the Fisher exact probability test used if the chi-square test condition was not met. The Mann–Whitney test was used to assess continuous variables. A two-tailed test level of α = 0.05 was adopted. Fine-Gray regression analysis was conducted to objectively evaluate the cumulative incidence of SCM. Non-SCM all-cause deaths were considered competing risk events to estimate the hazard ratio (HR) and 95% confidence interval (CI) for SCM occurrence.

The relative risk (RR) and 95% CI for the occurrence of SCM in BC patients who underwent RT vs those who did not were estimated using Poisson regression analysis, a model commonly used to describe the distribution of the number of rare (low probability) events. The SIR, also derived through Poisson regression, was defined as the ratio of the observed incidence of SCM in BC survivors in comparison to the incidence of CM in the general US population. This calculation was executed by utilizing the Multiple Primary SIR (MP-SIR) module of SEER*stat 8.4.1 software. In our analysis, both RR and the SIR were adjusted for age at the time of BC diagnosis and calendar year of diagnosis. To further assess the dynamic risk and incidence of RT-induced SCM, we calculated stratified RR and the SIR by latency time after BC diagnosis, age at BC diagnosis, and year of BC diagnosis.

To evaluate the prognosis of SCM, we calculated the 10-year OS for SCM vs only primary cutaneous melanoma (OPCM) using the Kaplan–Meier method. The *P* value was derived using the log-rank test (log-rank). OS was defined as the time from the diagnosis of SCM to death from any cause. The OPCM is defined as a patient who has had only CM and no other cancer diagnosis in their lifetime. To minimize potential survival comparison biases, propensity score matching (PSM) was implemented.

## Results

### Baseline distribution characteristics

A total of 520,977 BC patients met the screening criteria. The detailed flowchart is shown in Figure S1. The median age of clinic attendees was 60 years (IQR, 50–70). Most of the patients, 435,795 (83.6%) were white, 517,653 (99.6%) were female, and 3,324 (0.6%) were male. RT group comprised 243,676 (46.8%) patients, and 277,301 (53.2%) patients were in the NRT group. Table 1 provides a summary of the baseline characteristics of the BC patients based on the treatment modality (baseline clinicopathologic characteristics of the BC patients are supplemented to Table S1).

**Table 1:** Comparisons of baseline characteristics of patients with BC and SCM by treatment modality

	**All BC patients, No. (%)**			**All SCM patients, No. (%)**		
**Characteristics**	**NRT**	**RT**	***P***	**NRT**	**RT**	***P***
Age at BC diagnosis, median (IQR), years	62 (50–73)	59 (49–68)	**<0.001^a^**	59 (49–69)	59 (49–68)	0.894^a^
Age at BC diagnosis, years						
20–49	66,467 (24.0)	62,583 (25.7)	**<0.001^b^**	253 (27.1)	241 (25.5)	0.0677^b^
50–69	121,820 (43.9)	128,598 (52.8)		453 (48.6)	507 (53.7)	
≥70	89,014 (32.1)	52,495 (21.5)		226 (24.2)	196 (20.8)	
Year of BC diagnosis, median (IQR)	1995 (1886–2006)	2004 (1997–2011)	**<0.001^a^**	1993 (1986–2002)	2001 (1995–2007)	**<0.001** ^a^
Year of BC diagnosis						
1975–1984	60,568 (21.8)	14,232 (5.8)	**<0.001^b^**	196 (21.0)	59 (6.3)	**<0.001^b^**
1985–1994	76,724 (27.7)	34,816 (14.3)		311 (33.4)	172 (18.2)	
1995–2004	64,094 (23.1)	74,262 (30.5)		240 (25.8)	389 (41.2)	
≥2005	75,915 (27.4)	120,366 (49.4)		185 (19.8)	324 (34.3)	
Race						
White	235,767 (85.0)	200,028 (82.1)	**<0.001^b^**	912 (97.9)	927 (98.2)	0.222^b^
Black	22,498 (8.1)	21,797 (8.9)		10 (1.1)	4 (0.4)	
Other	19,036 (6.9)	21,851 (9.0)		10 (1.1)	13 (1.4)	
Tumor grade						
Grade I/II	109,305 (39.4)	137,859 (56.6)	**<0.001^b^**	363 (38.9)	560 (59.3)	**<0.001^b^**
Grade III/I V	71,121 (25.6)	71,188 (29.2)		220 (23.6)	223 (23.6)	
Unknown	96,875 (34.9)	34,629 (14.2)		349 (37.4)	161 (17.1)	
Tumor stage						
Localized	183,828 (66.3)	159,832 (65.6)	**<0.001^b^**	631 (67.7)	649 (68.8)	0.662^b^
Regional	93,473 (33.7)	83,844 (34.4)		301 (32.3)	295 (31.3)	
Tumor site						
LI	13,238 (4.8)	14,014 (5.8)	**<0.001^b^**	37 (4.0)	64 (6.8)	**<0.001^b^**
LO	19,120 (6.9)	17,494 (7.2)		60 (6.4)	70 (7.4)	
UI	25,611 (9.2)	29,469 (12.1)		73 (7.8)	126 (13.3)	
UO	93,786 (33.8)	91,298 (37.5)		330 (35.4)	341 (36.1)	
CEN	20,966 (7.6)	12,757 (5.2)		71 (7.6)	48 (5.1)	
Other	104,580 (37.7)	78,644 (32.3)		361 (38.7)	295 (31.3)	
Laterality						
Left	141,427 (51.0)	123,249 (50.6)	**<0.001^b^**	485 (52.0)	481 (51.0)	0.672^b^
Right	135,619 (48.9)	120,367 (49.4)		447 (48.0)	463 (49.0)	
Other	255 (0.1)	60 (0.0)				
Tumor histology						
Ductal	201,961 (72.8)	188,182 (77.2)	**<0.001^b^**	654 (70.2)	703 (74.5)	**0.007^b^**
Lobular	22,570 (8.1)	19,645 (8.1)		87 (9.3)	90 (9.5)	
Ductal and lobular	13,698 (4.9)	13,392 (5.5)		56 (6.0)	63 (6.7)	
Other	39,072 (14.1)	22,457 (9.2)		135 (14.5)	88 (9.3)	
Chemotherapy						
Yes	68,464 (24.7)	100,447 (41.2)	**<0.001^b^**	223 (23.9)	357 (37.8)	**<0.001^b^**
No/Unknown	208,837 (75.3)	143,229 (58.8)		709 (76.1)	587 (62.2)	
Follow-up time, median (IQR), months	121 (58–219)	112 (56–191)	**<0.001^a^**			
Latency, median (IQR), months	103.5 (48–173)	81.5 (40–144)	**<0.001^a^**			
Patients who developed SCM	932 (0.3)	944 (0.4)	**0.002^b^**			

^a^*P* value was calculated using the Mann–Whitney test for continuous; ^b^*P* value was calculated using X^2^ test for categorical variables; Significant *P* values are in bold. BC: Breast cancer; SCM: Second cutaneous melanoma; IQR: Interquartile range; LI: Lower inner quadrant of breast; LO: Lower outer quadrant of breast; UI: Upper inner quadrant of breast; UO: Upper outer quadrant of breast; CEN: Central portion quadrant of breast; RT: Radiation therapy; NRT: Non-radiation therapy.

BC patients who developed SCM after a 1-year latency period numbered 932 (0.3%) in the NRT group and 944 (0.4%) in the RT group. The median follow-up periods for SCM patients in the RT and NRT groups were 112 (IQR, 56–191) months and 121 (IQR, 58–219) months, respectively. There were significant differences between the two groups regarding the year of BC diagnosis, tumor grade, tumor site, chemotherapy, and clinicopathologic characteristics (Table 1 and Table S1).

### Cumulative and standardized incidence ratio (SIR) of second cutaneous melanoma (SCM)

**Figure 1.**
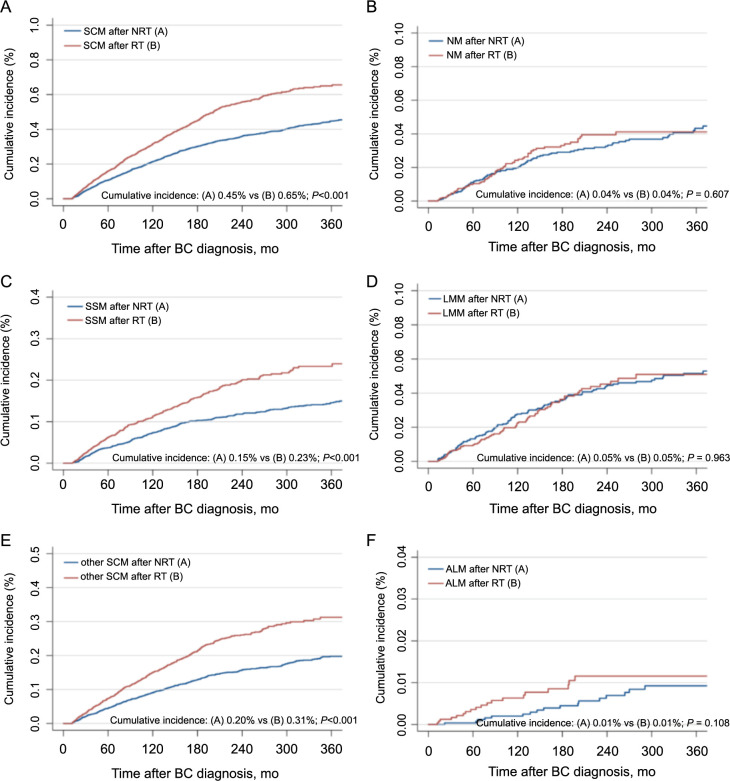
**Comparisons of the cumulative incidence of SCM in BC survivors (A) between patients who received RT and those who did not receive RT and (B-F) SCM's pathological subtypes between patients who received RT and those who did not receive RT.** Other SCMs indicate other SCM subtypes which include those not otherwise specified, except for the pathologic subtype of cutaneous melanoma described above. *P* values were calculated with the Fine-Gray test. RT: Radiation therapy; SCM: Second cutaneous melanoma; SSM: Superficial spreading melanoma; NM: Nodular melanoma; LMM: Lentigo maligna melanoma; ALM: Acral lentiginous melanoma; NRT: Non-radiation therapy.

The 30-year cumulative incidence of SCM patients was lower in the NRT group (0.45%) than in the RT group (0.65%) (*P* < 0.001), as illustrated in Figure 1A. In addition to analyzing overall SCM, we also conducted a specific analysis of SCM subtypes. The results showed that both SSM (30-year cumulative incidence of 0.23% vs. 0.15%; *P* < 0.001) and other SCMs (30-year cumulative incidence of 0.31% vs. 0.2%; *P* < 0.001) were significantly more prevalent in the RT. No significant differences in NM, LMM, and ALM incidences were found (Figure 1).

**Figure 2.**
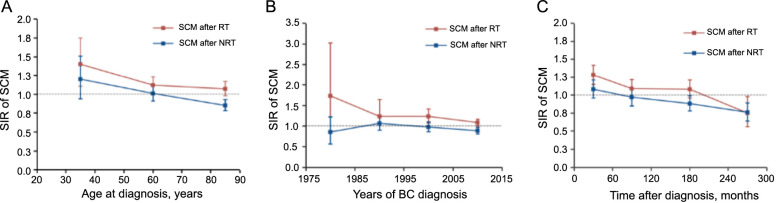
**Dynamic SIR for SCM in (A) the age-SIR plot, (B) diagnosis time-SIR plot and (C) the latency-SIR plot; Adjusted SIR and 95% CI of developing SCM in patients treated with RT versus the US general population are plotted, as well as patients treated without RT vs the US general population, and the incidence in the background US population is represented by the black line (at *y* ═ 1).** The detailed data of SIR are shown in Table S5. SCM: Second cutaneous melanoma; SIR: Standardized incidence ratio; RT: Radiation therapy; CI: Confidence interval; NRT: Non-radiation therapy; BC: Breast cancer.

To assess the risk of developing SCM in BC patients, we also calculated the SIR. The RT group exhibited a significantly higher risk of developing SCM than the general US population (SIR, 1.12; 95% CI, 1.05–1.19; *P* < 0.05). Additionally, we estimated the SIR for SCM stratified by age at diagnosis, year of diagnosis, and diagnostic latency (Figure 2A–2C).

### Risk of SCM occurring with radiation therapy (RT)

**Figure 3.**
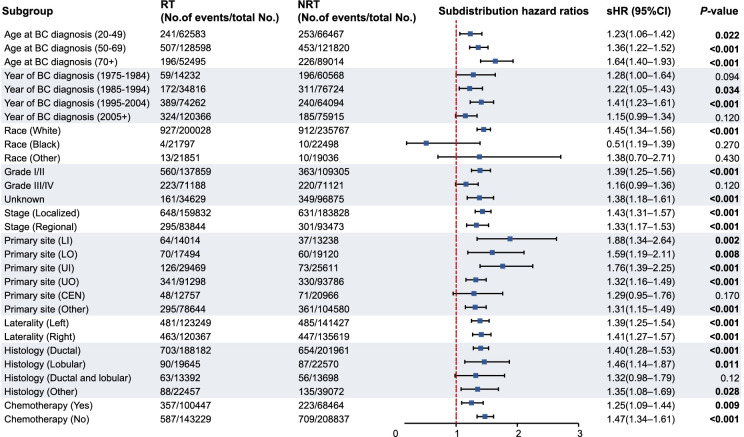
**Subgroup analyses of competing regression for the risk of developing second cutaneous melanoma**. The other sites of BC include axillary tail of breast and overlapping lesion of breast. Significant *P* values are in bold. BC: Breast cancer; RT: Radiation therapy; NRT: Non-radiation therapy; UO: Upper outer quadrant of breast; UI: Upper inner quadrant of breast; LI: Lower inner quadrant of breast; LO: Lower outer quadrant of breast; CEN: Central portion quadrant of breast; sHR: Subdistribution hazard ratio; CI: Confidence interval.

Univariate competing risk regressions were evaluated for the subdistribution HRs related to RT by employing the factors presented in the baseline table (shown in Table S2). Univariate analyses revealed *P* values of less than 0.05 for all variables except tumor site, tumor laterality, and chemotherapy. In multivariable analyses, age at diagnosis, calendar year of diagnosis, race, tumor histology, nodal stage, molecular subtype, and RT for BC were found to be more significantly associated with the risk of developing SCM (Table 2). In addition, RT was found to be an independent risk factor for developing SCM in BC survivors (HR, 1.24; 95% CI, 1.14–1.34, *P* < 0.001). Subgroup analyses indicated a statistically significant risk of developing SCM after RT for almost all subgroups (Figure 3).

**Table 2:** Multivariable computing risk regression analysis of developing SCM risk in BC patients

**Characteristics**	**Multivariable competing risk regression**	
	**sHR (95% CI)**	***P***
Age, per year	0.99 (0.99–0.99)	**<0.001**
Diagnosis, per year	1.02 (1.01–1.02)	**<0.001**
Race		
White	1	
Black	0.08 (0.05–0.12)	**<0.001**
Other	0.14 (0.10–0.20)	**<0.001**
Tumor histology		
Ductal	1	
Ductal and lobular	1.14 (0.97–1.34)	0.180
Lobular	1.19 (1.04–1.37)	**0.036**
Other	1.01 (0.89–1.14)	0.930
Radiotherapy		
No	1	
Yes	1.24 (1.14–1.34)	**<0.001**
Nodal stage		
N0	1	
N1	1.00 (0.82–1.24)	0.970
N2	0.78 (0.48–1.24)	0.370
N3	0.47 (0.27–0.82)	**0.024**
Unknown	0.52 (0.31–0.86)	**0.032**
Molecular subtype		
HR-/HER2- (Triple negative)	1	
HR-/HER2+ (HER2 enriched)	1.86 (0.92–3.76)	0.150
HR+/HER2- (Luminal A)	1.72 (1.07–2.77)	0.059
HR+/HER2+ (Luminal B)	2.11 (1.21–3.68)	**0.026**
Unknown	1.68 (1.06–2.67)	0.066

Fine-Gray competing risk regression analyses were used to calculate the HR and 95% CI for SCM in BC patients treated with RT versus patients not treated with RT. Covariables that are significant in univariable competing risk regression analysis (*P* < 0.05) are included in the multivariable analysis (Table S2). Significant *P* values are in bold. The other sites of BC include axillary tail of breast and overlapping lesion of breast. HR: Hormone receptor; HER2: Human epidermal growth factor receptor 2; sHR: Subdistribution hazard ratio; CI: Confidence interval; BC: Breast cancer; SCM: Second cutaneous melanoma; RT: Radiation therapy.

### SCM survival outcomes

**Figure 4.**
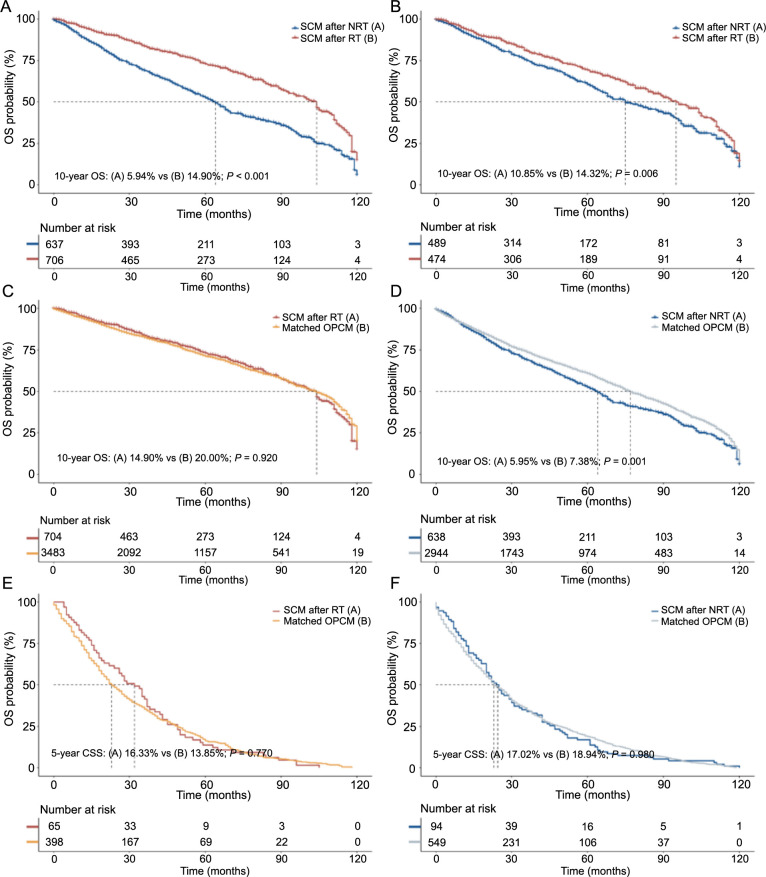
**Survival outcome of the SCM**. (A) Survival comparison between BC patients who developed SCM after RT and BC patients who developed SCM after NRT (before PSM) and (B) between BC patients who developed SCM after RT and BC patients who developed SCM after NRT (after PSM at a ratio of 1:1); SCM patients with RT (C and E) and without RT (D and F) were matched with OPCM patients based on propensity scores at a ratio of 1:5, and survival analysis was performed. The variables matched for PSM included age at SCM diagnosis, year of SCM diagnosis, race, stage, site, histology of SCM and type of treatment for SCM. Tables S8 and S9 reveal the complete patient characteristics of SCM and OPCM before and after PSM. BC: Breast cancer; RT: Radiation therapy; SCM: Second cutaneous melanoma; OPCM: Only primary cutaneous melanoma; PSM: Propensity score matching; OS: Overall survival; CSS: Cancer specific survival; NRT: Non-radiation therapy.

We compared the survival rates of patients with developing SCM who underwent RT for BC with those who did not. As illustrated in Figure 4A, the 10-year survival rate in the RT group was significantly higher than that in the NRT group (14.90% vs 5.94%, *P* < 0.001). After adjusting for the propensity to develop OPCM, there were no significant differences in 10-year OS or 5-year CSS between SCM and OPCM in the RT groups (Figure 4C and 4E). However, patients with SCM who did not receive RT for BC had a significantly lower 10-year OS than OPCM patients (5.95% vs. 7.38%, *P* ═ 0.001). There was no significant difference in 5-year CSS (Figure 4D and 4F).

## Discussion

To our knowledge, this is the first large population-based study designed to investigate the risk of developing SCM in BC survivors and to assess SCM survival outcomes. First, the BC patients who underwent RT had a higher cumulative incidence of SCM compared to those who did not. Secondly, multivariate competing risks regression analysis showed a significant association between RT and the development of SCM. Additionally, the rate of SCMs among BC patients receiving RT was higher than that of the general US population. Therefore, RT is considered an independent risk factor for SCM in BC patients. However, the risk of developing SCM after RT decreases with increasing latency and age, meaning that younger and middle-aged patients treated with radiation have a relatively higher risk than older patients. The SCM risk associated with RT stabilized in 1995 and then declined slightly. Finally, significant survival differences were also observed between SCM patients who had received RT for BC and those who developed SCM without RT.

Baseline characteristics shown in Table 1 reveal a notable variation in the year of diagnosis between the RT and NRT groups. Notably, there was a gradual increase in the number of patients who underwent RT after 1985. This trend coincides with the proliferation of RT over the past two decades. In line with previous research [10, 11], our analysis indicates that RT constitutes an independent risk factor for SCM. Experimental studies have demonstrated that ionizing radiation has the capacity to cause DNA damage, change cell cycle regulation, and activate or repress transcriptional mechanisms that are needed for DNA repair [12]. Moreover, RT may impair the patient’s immune system, particularly during axillary lymph node radiation, and this effect may be dependent on the tumor’s location within the body. As a result, RT might elevate melanoma mutations, potentially leading to the emergence of CM. There is limited evidence to support the idea that gut microbes may contribute to the pathogenesis and treatment of malignant melanoma [13]. RT has the potential to disrupt the composition of the gut microbiota, leading to gut microbiota dysbiosis and potentially impacting tumorigenesis and progression [14].

As listed in the literature in Table S10, previous studies estimating the risk of developing SCM in BC survivors have been controversial. These conflicting results seem to be explained by sample size, duration of follow-up, choice of latency period, and study methodology. Kirova et al. [15], investigating the risk of second malignancies in BC survivors receiving different treatment modalities, followed 16,705 patients with nonmetastatic BC and found that women treated for BC had an increased risk of second malignancies compared with the general population and that the increase may have been related to adjuvant therapy, but an increased risk of malignant melanoma was not observed. Schaapveld et al. [16], in assessing the risk of developing secondary non-breast cancer (SNBC) in a cohort of treated BC patients, did not observe a risk of RT associated with the development of SCM, although chemotherapy was observed to be associated with an increased risk of melanoma in patients over 50 years of age. Roychoudhuri et al. [17] compared the incidence of second primary cancers in patients who underwent breast RT and those who did not; 64,782 patients were included, a latency period of one year was selected, and there were no significant differences in SCM incidence in the RT cohort compared to the NRT group cohort at any time during follow-up. In conclusion, although all of the above studies were analyzed using large population-based cancer registries, the data were collected before 2000, and only a small number of patients with SCM were observed in the studies; the small sample sizes with limited statistical power and the lack of selection of an appropriate latency period make the results less convincing.

It is also possible that statistical methods differ across studies. Three main methods have been used to assess the risk of developing SCM after BC: (1) the SIR calculated based on Poisson distribution; (2) Cox regression; and (3) the log-rank test. Competing risk models for evaluating the risk of developing SCM were not utilized in the literature reviewed. Survival analysis utilizing the Fine-Gray competing risk model [18, 19] is a superior approach for analyzing survival data, as it allows for the endpoints to be categorized into multiple categories, distinguishing between the time of competition and the time of the outcome being studied and removing the impact of competition on prognostic studies.

In this study on the longitudinal incidence of SCM, we adjusted for age group, BC diagnostic latency, and year of diagnosis and found that the risk of developing SCM after RT stabilized after 1990, with a small decrease after 2000, which may be explained by innovations in radiation modalities. Conventional two-dimensional RT is limited in conditions, and the irradiated area may include many normal tissues surrounding the tumor, thus increasing the risk of treatment. To overcome the problem of excessive irradiation of normal tissues, three-dimensional conformal RT (3D-CRT) was proposed in the late 1990s, and in the early 2000s, revolutionary intensity-modulated RT (IMRT) based on 3D-CRT was developed, which can make the dose distribution in the field more uniform and reasonable [20]. The development of precision RT techniques has reduced the amount of radiation exposure in the surrounding normal tissues, improved the local control rate of tumors, and reduced patient collateral damage. However, other studies [9] have suggested that IMRT may expose patients to higher levels of radiation leakage than conventional 3D-CRT, leading to a higher incidence of second primary cancers. The effect of RT on the incidence of second cancers is unclear and may require prospective studies with 10 or more years of follow-up.

To date, there are no studies on the prognosis of RT-associated SCM. This is a key clinical issue in SCM because the prognosis may be heterogeneous, and different genetic pathways may be induced after radiation exposure. In our study, there was a significant difference in the survival rate of SCM patients between the RT and NRT groups. We further performed survival analysis to evaluate the prognosis of SCM in the RT and NRT groups with matched OPCM. The results showed that in the RT group, there was no significant difference in OS and CSS compared with OPCM; however, in the NRT group, the prognosis was poor compared with OPCM. Radiotherapy for BC has a dual effect on SCM: it increases the risk but does not worsen the prognosis. The initial treatment modality of BC has a weak positive effect on survival in the early stages of SCM after diagnosis. This may be because most tumors in BC patients treated with radiotherapy for second CMs are at an early stage, and surgical excision is still the standard of care with a good prognosis.

The study has several strengths, including long-term follow-up to identify potential SCM patients, a large observational population with relatively homogeneous treatment exposures identified from the SEER database, and risk and prognostic comparisons performed. We must also consider the limitations of this study, which include the following: (1) The SIR for SCM was more significant in BC patients who underwent RT with latency within the first five years, possibly because patients diagnosed and treated for BC receive more frequent surveillance, making it easier for patients to detect and report cutaneous malignant melanoma than in the general population; thus, the increased incidence of SCM appears to be due to surveillance bias; (2) The lack of randomization of primary BC treatment is also subject to potential bias, making it impossible to adjust for all factors between the two types of treatment. Instead, we adjusted for all confounders using a multivariate competing risk model to reduce the potential bias associated with a lack of randomization; (3) The SEER database only records information on the initial treatment of BC patients, and we were unable to determine the associations of RT modality, RT dose, and the number of RT administrations with the risk of developing SCM. In addition, it is not known whether BC patients underwent delayed RT at follow-up, which may have misclassified patients in the sub-RT group as the NRT group. However, this limitation is unlikely to affect our main conclusions and only reflects an underestimation of the increased risk due to RT; (4) The development of SCM is not only related to RT but may also be influenced by other important risk factors, such as lifestyle, genetic background [21], environmental factors, and other cancer-related treatments [9].

## Conclusion

This study suggests that while RT for BC is associated with a slightly increased SCM risk, the benefits of RT for BC survival outweigh the risk of RT-induced second cancers. This information could guide the management and follow-up of BC patients developing SCM post-RT and assist in future surveillance planning.

## Supplemental data

Supplementary data can be found at the following link: https://www.bjbms.org/ojs/index.php/bjbms/article/view/10029/3148
